# Antifibrotic Mechanism of Piceatannol in Bleomycin-Induced Pulmonary Fibrosis in Mice

**DOI:** 10.3389/fphar.2022.771031

**Published:** 2022-06-07

**Authors:** Hanjing Sheng, Gang Lin, Shengxian Zhao, Weibin Li, Zhaolin Zhang, Weidong Zhang, Li Yun, Xiaoyang Yan, Hongyu Hu

**Affiliations:** ^1^ Xingzhi College, Zhejiang Normal University, Lanxi, China; ^2^ Xiamen University, Xiamen, China; ^3^ College of Science and Technology, Ningbo University, Cixi, China; ^4^ The Key Laboratory for Endocrine-Related Cancer Precision Medicine of Xiamen, The Cancer Center and the Department of Breast-Thyroid Surgery, Xiang’ an Hospital of Xiamen University, School of Medicine, Xiamen University, Xiamen, China; ^5^ Affiliated Jinhua Hospital, Zhejiang University School of Medicine, Jinhua, China

**Keywords:** autophagy, TGF-β1, myofibroblasts, pulmonary fibrosis, piceatannol

## Abstract

**Background:** Idiopathic pulmonary fibrosis (IPF) is a progressive and fatal interstitial lung disease characterized by myofibroblast accumulation and extracellular matrix deposition, which lead to irreversible damage of the lung’s architecture and the formation of fibrotic lesions. IPF is also a sequela in serious patients with the coronavirus disease 2019 (COVID-19). The molecular mechanisms under pulmonary fibrosis remain unclear, and there is no satisfactory treatment currently available. Piceatannol (PIC) is a naturally occurring resveratrol analog found in a variety of dietary sources such as grapes, passion fruit, and white tea. It has been reported to inhibit liver fibroblast growth and exhibited various antitumor activities, although its role in pulmonary fibrosis has not been established yet. In the present study, we evaluated the anti-fibrotic role of PIC in bleomycin (BLM)-induced pulmonary fibrosis in mice.

**Methods:** Mice with BLM-induced pulmonary fibrosis were treated with PIC, and fibrotic changes were measured by hematoxylin-eosin (H&E) staining and hydroxyproline assay. Luciferase assay, Western blot assay, histological analysis, and immunofluorescence staining were used to evaluate the effect of PIC on fibroblast activation and autophagy in mouse embryonic fibroblast cells (NIH-3T3) and human lung fibroblast cells (HFL1). The anti-fibrotic mechanisms of PIC were either confirmed *in vivo*.

**Results:** Our results showed that PIC significantly alleviated the bleomycin-induced collagen deposition and myofibroblast accumulation. *In vitro* and *in vivo* studies indicated that PIC plays a role in activating autophagy in the process of anti-fibroblast activation. Further mechanism studies demonstrated that PIC can promote autophagy *via* inhibiting the TGF-β1-Smad3/ERK/P38 signaling pathway, which leads to a decreased number of activated myofibroblasts.

**Conclusion:** Our study demonstrated for the first time that PIC possesses the protective effects against bleomycin-induced pulmonary fibrosis due to the direct pulmonary protective effects which enhance the effect of autophagy *in vitro* and *in vivo* and finally leads to the decreased number of activated myofibroblasts. PIC may serve as a candidate compound for pulmonary fibrosis therapy and attenuates the sequelae of SARS-COV-2 pulmonary fibrosis.

## Graphical Abstract

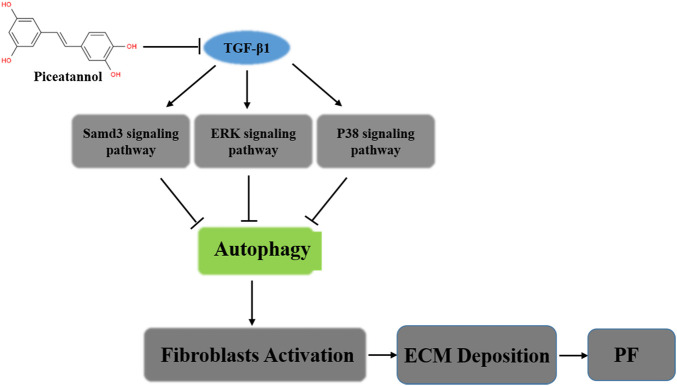

## Introduction

Idiopathic pulmonary fibrosis (IPF) is the most common type of idiopathic interstitial pneumonia (Dong et al., 2015), which is a progressive and fatal lung disease with a high mortality rate (Selman et al., 2011). The mean survival of IPF patients after diagnosis ranges from 2 to 3 years. The precise pathomechanisms of IPF remain incompletely understood. It has been speculated that many microlesions from outside lead to destruction of alveolar epithelial cells, resulting in a disordered wound healing process that induces massive hyperplasia of active fibroblasts and excessive collagen deposition in the pulmonary interstitial and alveolar spaces (Fernandez and Eickelberg, 2012). The large amount of collagen deposited in the interstitial lung destroys the normal alveolar structure, causing loss of respiratory function in patients with IPF, eventually leading to respiratory failure and death (Karsdal et al., 2015). The extracellular matrix (ECM) is a complex grid consisting of multiple proteins, most of which play an important role in maintaining the normal function of the body (Wynn and Thomas, 2007). The excessive and abnormal accumulation of extracellular matrix (ECM) components is likely to lead to fibroproliferative diseases (Matthew et al., 2015).

From the perspective of epidemiology, viral immunology, and current clinical research, pulmonary fibrosis may become one of the complications of patients with the current coronavirus disease 2019 (COVID-19) (Li et al., 2020). A total of 311 patients with SARS-CoV-2 infection on the research network were identified, and 251 patients (0.08%) carried a diagnosis of IPF (Kang et al., 2020). Therefore, pulmonary fibrosis is likely to be one of the major sequelae in COVID-19 patients (Kang et al., 2020). In the case of COVID-19, the conclusion from clinical studies is that excessive production of pro-inflammatory cytokines leads to ARDS aggravation, extensive and multiple organ damage, and the formation of pulmonary fibrosis, organ failure, and even death. (Alam et al., 2020). So far, there has been no effective therapy for IPF treatment except for lung transplants. Therapies such as immunosuppressants (e.g., cyclophosphamide) are limited by low efficacy and severe side effects. Recently, the FDA approved two new drugs to treat IPF. These drugs can effectively alleviate the symptoms of decreased lung function in patients but could not prolong the survival of patients (Ogura et al., 2015; Helen et al., 2016). Therefore, new therapeutic drugs with improvement in the treatment of IPF efficacy and the sequelae of SARS-COV-2 pulmonary fibrosis effects are urgently needed (Zhang et al., 2021).

As a unique medical treatment, traditional Chinese medicine (TCM) plays an important role in the treatment of IPF (Zhang et al., 2021). Piceatannol (PIC), as a TCM, is a polyphenolic analog of resveratrol that selectively inhibits the non-receptor tyrosine kinase-Syk (Kita et al., 2014; Abdelgawad et al., 2016). Previous studies reported that PIC showed inhibitory effects on various human cancer cells with less cytotoxicity, including prostate cancer, breast cancer, and hepatocellular carcinoma (Luft et al., 2008; Ko et al., 2012; Kwon et al., 2012). Except for the antitumor activity, PIC also exerts the ability to aid in hepatocyte protection and to guard against hepatic fibrosis induced by thioacetamide (TAA) intoxication (Shinya Mizuno, 2007; Torres-Hernandez et al., 2019). However, the mechanism responsible for PIC exerting the anti-fibrotic function in pulmonary fibrosis is poorly understood.

In the present study, we found that PIC could alleviate BLM-induced pulmonary fibrosis in mice and suppress the accumulation of important effector cells (myofibroblasts) in the pathogenesis of fibrosis, which suggested PIC may serve as a potential compound for IPF treatment.

## Materials and Methods

### Reagents and Antibodies

Antibodies against α-SMA, collagen type I, fibronectin, E-cadherin, vimentin, phospho-Smad3, phospho-p38, p-38, phospho-ERK1/2 (T202/Y204), and ERK1/2 were obtained from Cell Signaling Technology (Beverly, MA). Antibodies against LC3II and p-62 were obtained from Santa Cruz Biotechnology (Santa Cruz, CA). Anti-mouse immunoglobulin G and anti-rabbit immunoglobulin G fluorescent-conjugated secondary antibodies obtained were from LI-COR Biotechnology (Nebraska, United States). Dulbecco’s Modified Eagle Medium (DMEM) was obtained from Cytiva, United States. Fetal bovine serum (FBS) was obtained from Grand Island Biological Company (Gibco, United States). DAPI was obtained from Yeasen Biotech Co., Ltd. (Yeasen Shanghai). SB-431542 was obtained from MedChemExpress (MCE, United States). Piceatannol was from Chengdu Herbpurify Co., Ltd.

### Cell Culture

Mouse embryo fibroblast cells (NIH3T3), human lung fibroblasts (HFL1), and mouse fibroblast cells (Mlg) were obtained from ATCC. All cells were cultured in DMEM or 1,640 supplemented with 10% FBS and contained 10 μg/ml penicillin-streptomycin in 5% CO_2_ at 37°C in a humidified atmosphere.

### Immunofluorescence Staining

NIH-3T3 cells were seeded on glass slides at the bottom of a 24-well plate and cultured for 24 h by containing 10% serum medium. The slides were washed three times with a concentration of PBS (1×) and fixed in 4% paraformaldehyde for 15 min. Then, cells were permeabilized with 0.2% Triton X-100 for 20 min, then blocked with 5% BSA for 30 min to eliminate the non-specific background, and incubated with primary antibodies (α-SMA, 1:200; p-ERK, 1:100; p-p38, 1:100, p-Smad3, 1:200, LC3II, 1:100) at 4° overnight. Then, the cells were washed with PBS three times and incubated in the corresponding fluorescent secondary antibody for 2 h at room temperature. The nuclei were stained with DAPI. Representative micrographs were acquired using a laser scanning confocal microscope (Leica SP8, Germany).

### Quantitative Real-Time PCR

Total RNA was extracted from cells or tissues using TRIzol reagent, according to the manufacturer’s instructions. Gene expressions of α-SMA, collagen type I, fibronectin (FN), and CTGF were detected by quantitative real-time PCR using SYBR^®^ Green Real-Time PCR Master Mixes. Each measurement was repeated in triplicate and normalized to the levels of β-actin mRNA. The sequences of specific primer pairs are described as follows: β-actin forward, 5-AGG​CCA​ACC​GTG​AAA​AGA​TG-3 and reverse, 5-AGA​GCA​TAG​CCC​TCG​TAG​ATG​G-3; a-SMA forward, 5-CTA​TGA​GGG​CTA​GCC​TTG​CC-3 and reverse, 5-GCT​CAG​CAG​TAG​TAA​CGA​AGG​A-3; Col1a1 forward, 5-AGG​GCA​ACA​GCA​GGT​TCA​CTT​ACA-3 and reverse, 5-AGC​GGG​GGA​AGG​AGT​TAA​TGA​AAC-3; FN forward, CGG​TGG​CTG​TCA​GTC​AAA​G and reverse, AAA​CCT​CGG​CTT​CCT​CCA​TAA; CTGF forward, 5-CCC​TGA​CCC​AAC​TAT​GAT​GC-3 and reverse, 5-CCT​TAC​TCC​CTG​GCT​TTA​CG-3.

### Luciferase Assay

The NIH3T3 cell line with EM containing 10 stable pCAGA (12)-luc expressions was utilized to detect the TGF-β1 activity with high sensitivity. The cells were cultured in DMEM and 10% FBS for 24 h. Then, the cells were treated with 5 ng/ml TGF-β1 with or without PIC (PIC, 4 μM) in DMEM containing 0.1% FBS for 24 h. After washing with PBS, cells were harvested, and the luciferase activity of cell lysates was determined using a luciferase assay system, as described by the manufacturer (Promega). All assays were repeated in triplicate.

### Western Blot

The proteins were extracted from cells or tissue following standard protocols, as described previously (Ning et al., 2004). Membranes were incubated at 4°C overnight with primary antibodies. After incubation, membranes were washed thrice with PBST and incubated for 2 h with the anti-rabbit or anti-mouse HRP-conjugated secondary antibody. The blots were visualized using the ECL Plus chemiluminescent kit, according to the manufacturer’s instructions. Protein bands were quantified by ImageJ software (NIH, Bethesda, Maryland, United States).

### BLM-Induced Animal Model of Pulmonary Fibrosis

Male C57BL/6 mice (6–8 weeks, 20 mg) were purchased from Beijing Vital River Laboratory Animal Technology Co. (Beijing, China). All mice were housed and cared for in a pathogen-free facility at Zhejiang Normal University. All animal experiments were approved by the Animal Care and Use Committee at Zhejiang Normal University (Approval no. ZSDW202151201908006x). Twenty-four mice were divided into four groups with six animals per group according to body weight: the control group, bleomycin group, bleomycin + PIC group (10 mg/kg), and bleomycin + pirfenidone group (100 mg/kg). Mice were anesthetized with chloral hydrate (Sangon) and then intratracheally injected with bleomycin (Blenoxane, Nippon Kayaku Co., Ltd.) at a dose of 2.5 U/kg bodyweight for analysis of the fibrotic response. PIC was intragastrically administered daily for a week, beginning 7 days after bleomycin injury, and pirfenidone was used as the positive control. Control and model groups received an equal volume of vehicle (0.9% NaCl) using the same schedule and route of administration. On the 14th day after bleomycin injection, mice were euthanized with chloral hydrate by i.p. injection, hearts were perfused with PBS through the right ventricle until lungs were cleared of blood, and lungs were harvested for further analysis.

### Hydroxyproline Assay

Collagen contents in the right lungs were measured with a conventional hydroxyproline method (Jiang et al., 2004). The ability of the assay to completely hydrolyze and recover hydroxyproline from collagen was confirmed using samples containing known amounts of purified collagen.

### Confocal Microscopy and Transmission Electron Microscope

To determine the formation of autophagosomes and autolysosomes, the mRFP-GFP-LC3 (Sigma) plasmid was transfected into NIH-3T3 cells using Lipofectamine 2000 and incubated for 6 h. Cells were stimulated without or with TGF-β1 (5 ng/ml), followed by treatment with PIC (4 μM) for 24 h. Cells were fixed with 4% paraformaldehyde and stained with DAPI under permeabilizing conditions. Images were acquired using a laser scanning confocal microscope (Leica SP8, Germany).

For transmission electron microscopic (TEM) examination, cells were fixed in 2.5% glutaraldehyde overnight at 4°C, post-fixed with 1% osmium tetroxide for 1 h at RT, dehydrated, and embedded. The number of AVs per cell was examined under a transmission electron microscope (Hitachi, Japan).

### Histology and Immunohistochemistry

The left lungs were inflated with 0.5 ml of 10% neutral buffered formalin. The tissues were then fixed overnight, embedded in paraffin, and sectioned for staining with hematoxylin and eosin to assess the degree of fibrosis. Slides stained with trichrome were examined under high power and scored for a total of 10 random fields per specimen. Digitized images were analyzed by Image-Pro Plus 6.0 software (Media Cybernetics). The overall and fibrotic areas of the lung were outlined, the pixels of total vs. fibrotic tissue were summed over each lung, and a percentage was obtained. Immunohistochemistry was performed, as previously described (Geng et al., 2011). In brief, 5-μm lung sections were deparaffinized and rehydrated. After the antigen was recovered by high-pressure heating with citrate buffer (MaximBio), sections were incubated with different antibodies at 4°C overnight. After being washed with PBST three times, sections were incubated with HRP-polymer secondary antibodies (MaximBio) for 15 min and then stained with DAB solution (MaximBio). The nucleus was stained with hematoxylin.

### Statistical Analysis

Data are expressed as the mean ± SEM. Differences in measured variables between the experimental and control groups were assessed by using Student’s t-tests. Results were considered statistically significant at *p < 0.05. SPSS software was used for statistical analysis.

## Results

### PIC Attenuates TGF-β1-Induced Fibroblast Activation and ECM Production in Fibroblasts *in Vitro*

**FIGURE 1:**
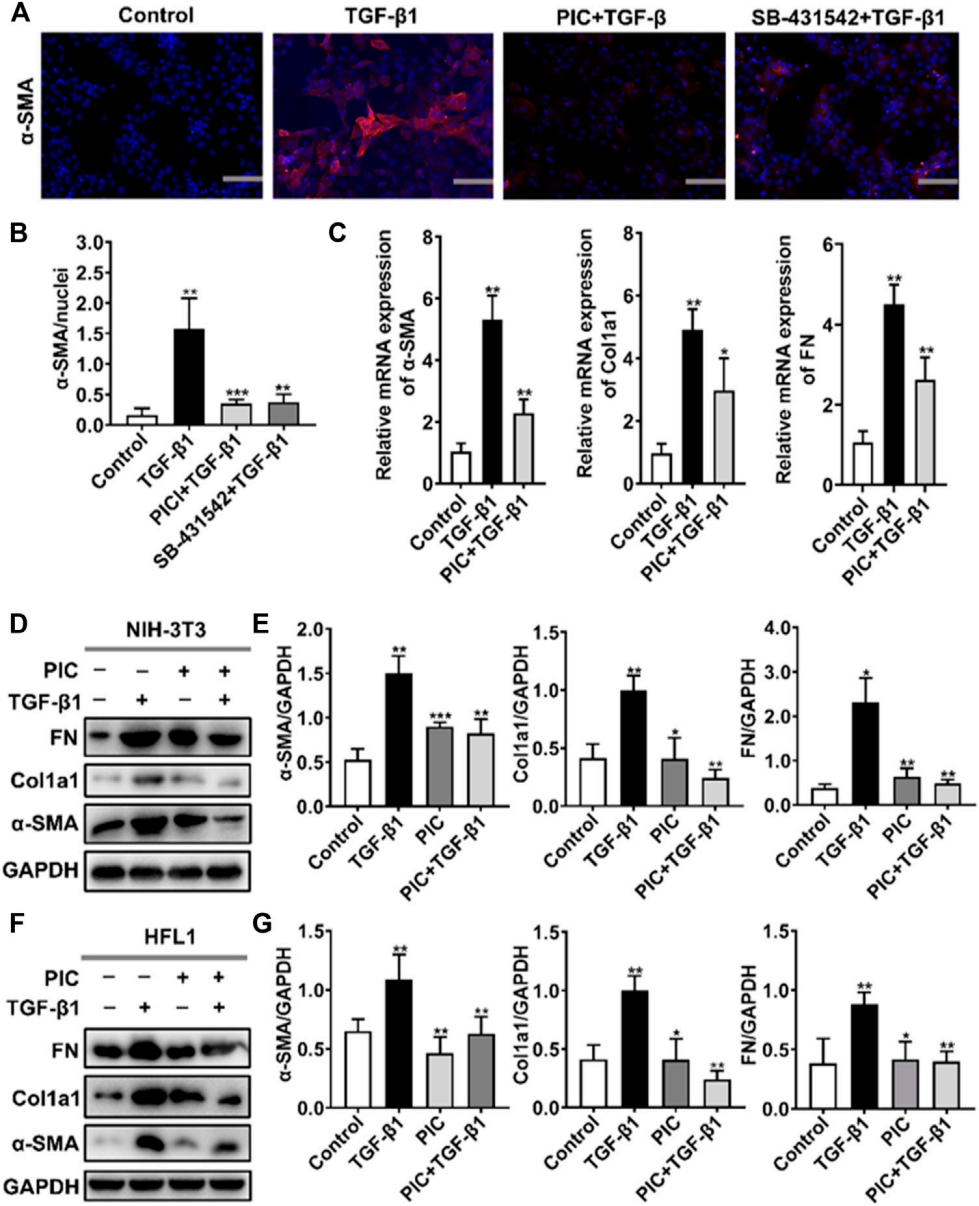
**(A)** NIH-3T3 cells were treated with TGF-β1 (5 ng/ml) and PIC (4 μM) or SB-431542 (10 μM) for 24 h. Immunofluorescence staining of polymerized α-actin stress fibers (nucleus, DAPI) in each group is shown. Scale bar, 60 µm. **(B)** Quantitative analysis of the α-SMA/nuclei fluorescence ratio in each group. **(C)** NIH-3T3 cells were treated with TGF-β1 (5 ng/ml) and PIC (4 μM) for 12 h. Relative expressions of α-SMA, Col1a1, and FN mRNA levels were detected by real-time quantitative PCR. β-actin was used as a control. **(D)** NIH-3T3 cells were treated with TGF-β1 (5 ng/ml) with or without PIC (4 μM) for 24 h. Western blot analysis showed protein expressions of α-SMA, Col1a1, and FN in cells. **(E)** Quantitative protein expression analysis of α-SMA, Col1a1, and FN compared with GAPDH in different groups. **(F)** HFL1 cells were treated with TGF-β1 (5 ng/ml) with or without PIC (4 μM) for 24 h. Western blot analysis showed protein expressions of α-SMA, Col1a1, and FN in cells. **(G)** Quantitative Western blot analysis of α-SMA, Col1a1, and FN compared with GAPDH in different groups. Data are expressed as mean ± SD (n = 3). *p < 0.05 and **p < 0.01.

To assess the role of PIC on myofibroblast activation *in vitro*, we established a cell model with TGF-β1 to induce fibroblast activation. A hallmark of the myofibroblast cell state is the high expression of α-SMA. Immunofluorescence assay showed that α-SMA fluorescence was enhanced after TGF-β1 stimulation, but PIC significantly decreased the fluorescence intensity of α-SMA. SB-431542 is a potent and selective inhibitor of ALK5/TGF-β type I receptor, and it can inhibit the activity of TGF-β1. The results showed that SB-431542 and PIC suppressed α-SMA fluorescence (Figures 1A,B). In addition, the α-SMA, Col1a1, and FN mRNA expression levels were also detected. TGF-β1 treatment significantly upregulated the expressions of mRNA of α-SMA, Col1a1, and FN, but PIC treatment significantly reduced the mRNA level of these reliable markers of myofibroblasts (Figure 1C). In addition, Western blot analysis showed that PIC treatment significantly reduced the protein level of α-SMA, Col1a1, and FN in TGF-β1-stimulated NIH-3T3 cells (Figures 1D,E). These results were also proved in HFL1 cells (Figures 1F,G). Collectively, these results suggest that PIC attenuates TGF-β1-induced fibroblast activation and ECM production in fibroblasts *in vitro*.

### PIC Attenuates TGF-β1-Induced Fibroblast Activation by Promoting Autophagy *in Vitro*

**FIGURE 2:**
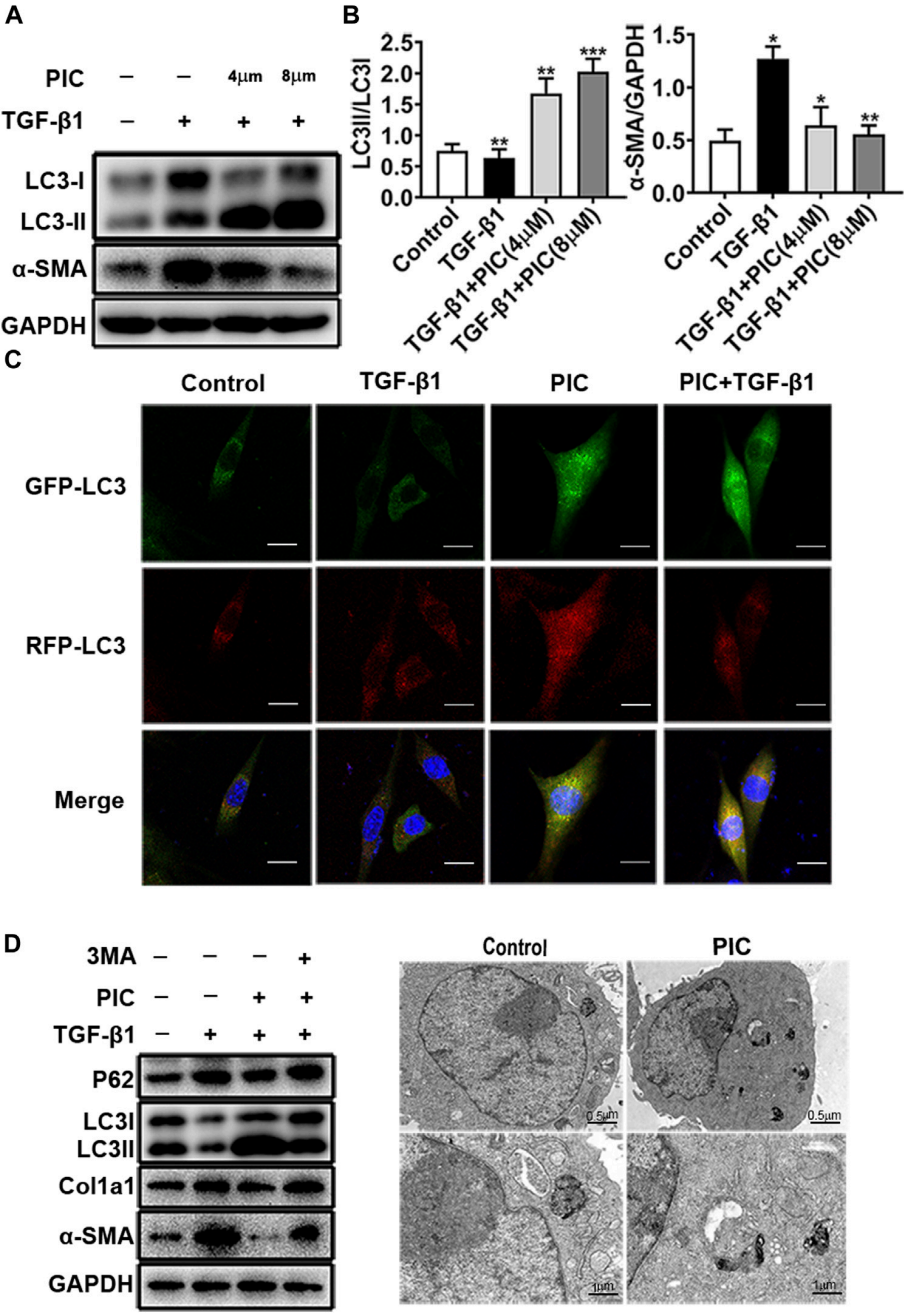
**(A)** Protein expressions of LC3I, LC3II, and α-SMA in NIH-3T3 cells treated with 5 ng/ml TGF-β1 with or without PIC (4 μM and 8 μM) for 24 h. **(B)** Quantitative Western blot analysis of LC3I/LC3II/α-SMA compared to GAPDH in different groups. **(C)** NIH-3T3 cells, transfected with the plasmid mRFP-GFP-LC3, were treated with PIC (4 μM) for 24 h in the existence or absence of stimulation by TGF-β (5 ng/ml). GFP and RFP were shown as LC3-positive cells and DAPI as nuclear. **(D)** Protein expressions of LC3I, LC3II, P62, Col1a1, α-SMA, and GAPDH in lung fibroblasts (NIH-3T3) treated with 5 ng/ml TGF-β1, 3 MA (2 mM) with or without PIC (4 μM) for 24 h. **(E)** Representative images of fibroblasts were treated with TGF-β1 and/or PIC (4 μM); then, autophagic vesicles (AVs) were analyzed by using a transmission electron microscope. The expression of protein was performed by ImageJ using GAPDH as a loading control. Data are expressed as mean ± SD, n = 3. *p < 0.05, **p < 0.01, and ***p < 0.001.

PIC was reported to induce autophagy in many cancer cells (Choi et al., 2015; Papandreou et al., 2015; Yusuke et al., 2017; Siedlecka-Kroplewska et al., 2019). In addition, autophagy plays an important role in pulmonary fibrosis and fibroblast activation (Patel et al., 2014). So to investigate whether autophagy was involved in the attenuation of fibroblast activation induced by PIC, protein levels of LC3I, LC3II, and α-SMA were analyzed by Western blot analysis. The results indicated that PIC significantly decreased α-SMA protein levels and upregulated the protein ratio of LC3II/LC3I in dose-dependent manners in NIH-3T3 cells treated with TGF-β1, indicating that PIC could induce autophagy in NIH-3T3 cells (Figures 2A,B). To further confirm the effects of PIC on autophagy in NIH-3T3 cells induced by TGF-β1, the mRFP-GFP-LC3 system, which is a typical tool to monitor autophagic flux based on the different PH stabilities of the EGFP and mPFP fluorescent proteins, was employed. The expression of mRFP-GFP-LC3 resulted in both green and red fluorescence (Masliah et al., 1990; Kimura et al., 2007). EGFP fluorescence is easily quenched in a low-pH environment, whereas mRFP is more stable in an acidic environment. Colocalization of EGFP and mRFP fluorescence (merged as yellow) indicates compartments that are not fused with acidic lysosomes. Therefore, both yellow and red punctate fluorescence will increase in the case of autophagy activation, whereas blockade in the degradation stage results in only yellow punctate fluorescence. After treatment of the cells with PIC with or without TGF-β1, red punctate fluorescence markedly increased, indicating PIC induced successful autophagy in the cells (Figure 2C). Next, 3-MA (autophagy inhibitor) was used to investigate whether PIC attenuated TGF-β1-induced fibroblast activation by promoting autophagy. The results indicated that 3 MA could significantly reverse autophagy induced by PIC in cells stimulated with TGF-β1 (Figure 2D). In addition, we also investigated whether 3-MA could affect the activation of fibroblasts. The results showed that 3-MA significantly increased the protein levels of α-SMA and Col1a1 in PIC-treated cells stimulated by TGF-β1 (Figure 2D). Furthermore, we assessed the activating autophagy of PIC and observed that transmission electron microscopy (TEM) also revealed more autophagic vesicles (AVs) containing engulfed organelles in PIC-treated fibroblasts (Figure 2E). Taken together, these results indicated that PIC could inhibit fibroblast activation by promoting autophagy.

### PIC Attenuates TGF-β1-Induced Fibroblast Activation by Enhancing Autophagy *via* the Smad3/ERK/P38 Signaling Pathway *in Vitro*

**FIGURE 3:**
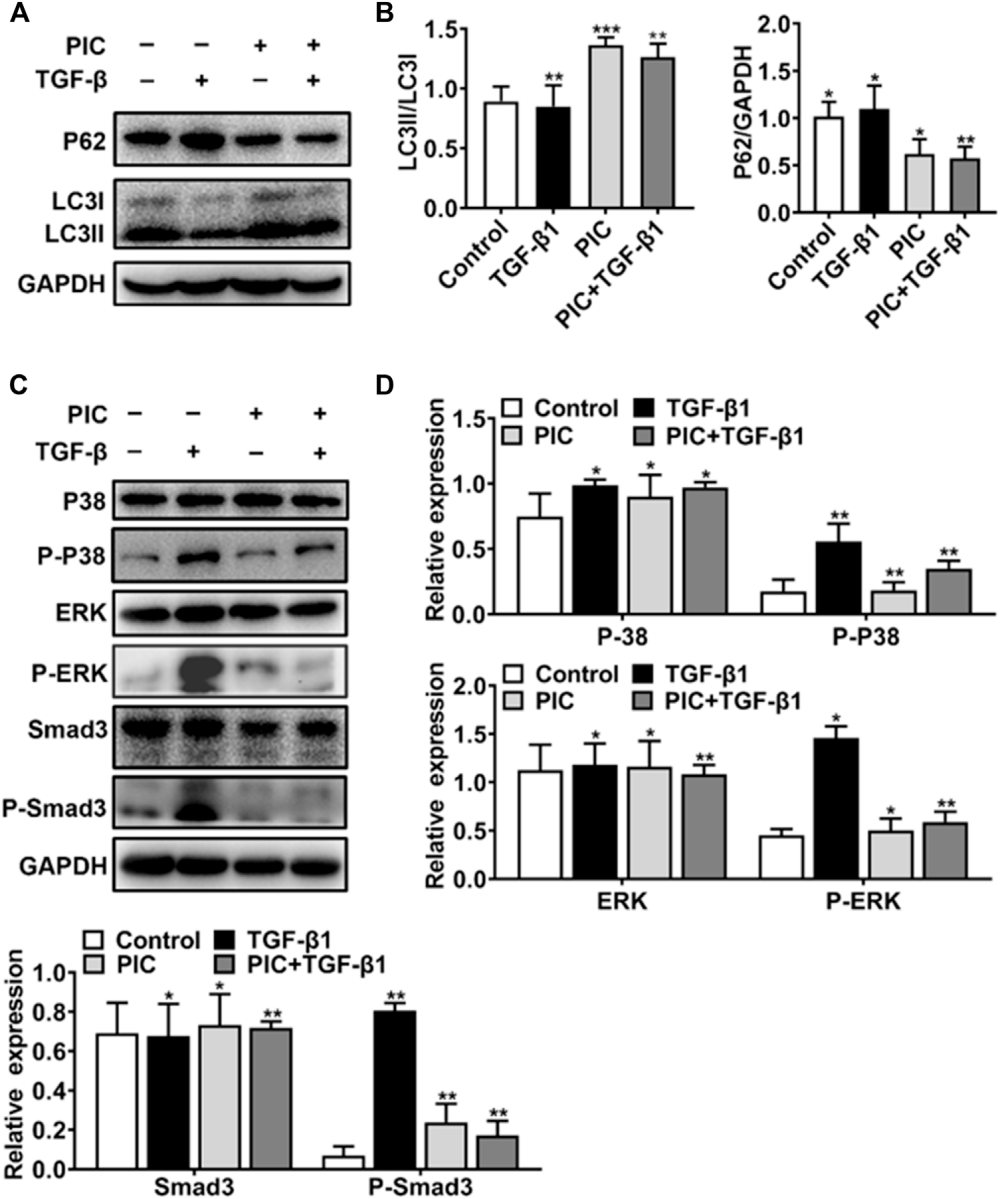
**(A)** Protein expressions of LC3I, LC3II, P62, and GAPDH in NIH-3T3 cells treated with 5 ng/ml TGF-β1 with or without PIC (4 μM) for 24 h **(B)** Quantitative Western blot analysis of α-SMA, LC3I, LC3II, and GAPDH in different groups. **(C)** Protein expressions of p-Smad3, Smad3, p-ERK, ERK, P-P38, and P38, in NIH-3T3 cells treated with 5 ng/ml TGF-β1 with or without PIC (4 μM) for 1 h. **(D)** Quantitative Western blot analysis of p-Smad3, Smad3, p-ERK, ERK, p-P38, and P38 in different groups. Data are expressed as mean ± SD, n = 3. *p < 0.05, **p < 0.01, and ***p < 0.001.

To further analyze the PIC’s mechanism of action, which activates autophagy and inhibits fibroblast activation, first, Western blot analysis showed that PIC treatment significantly accumulated the protein ratio of LC3II/LC3I and reduced the protein level of p62 in NIH-3T3 cells treated with TGF-β1 (Figures 3A,B). Also, luciferase assay was performed to investigate whether PIC inhibits fibroblast activation through influencing the TGF-β1-Smad3 signaling pathway. The results showed that PIC obviously inhibited the activation of the Smad3 signaling pathway in TGF-β1–stimulated cells (See supplementary data). Furthermore, the protein levels of phosphorylated Smad3 were analyzed by Western blot analysis. The results showed that PIC treatment significantly decreased the expression of phosphorylated Smad3. (Figures 3C,D). These results demonstrated PIC attenuates TGF-β1-induced fibroblast activation by inhibiting the TGF-β/Smad3 signaling pathway. Next, we investigated the effects of PIC on non-Smad signaling pathways including the MAPK pathways. PIC treatment significantly decreased the activation of p-ERK and p-p38 stimulated by TGF-β1 (Figures 3D,E). Based on the aforementioned results, we can conclude that PIC enhances autophagy *via* the Smad3/ERK/P38 signaling pathway and inhibits TGF-β–induced fibroblast activation *in vitro*.

### PIC Attenuates Bleomycin-Induced Pulmonary Fibrosis in Mice

**FIGURE 4:**
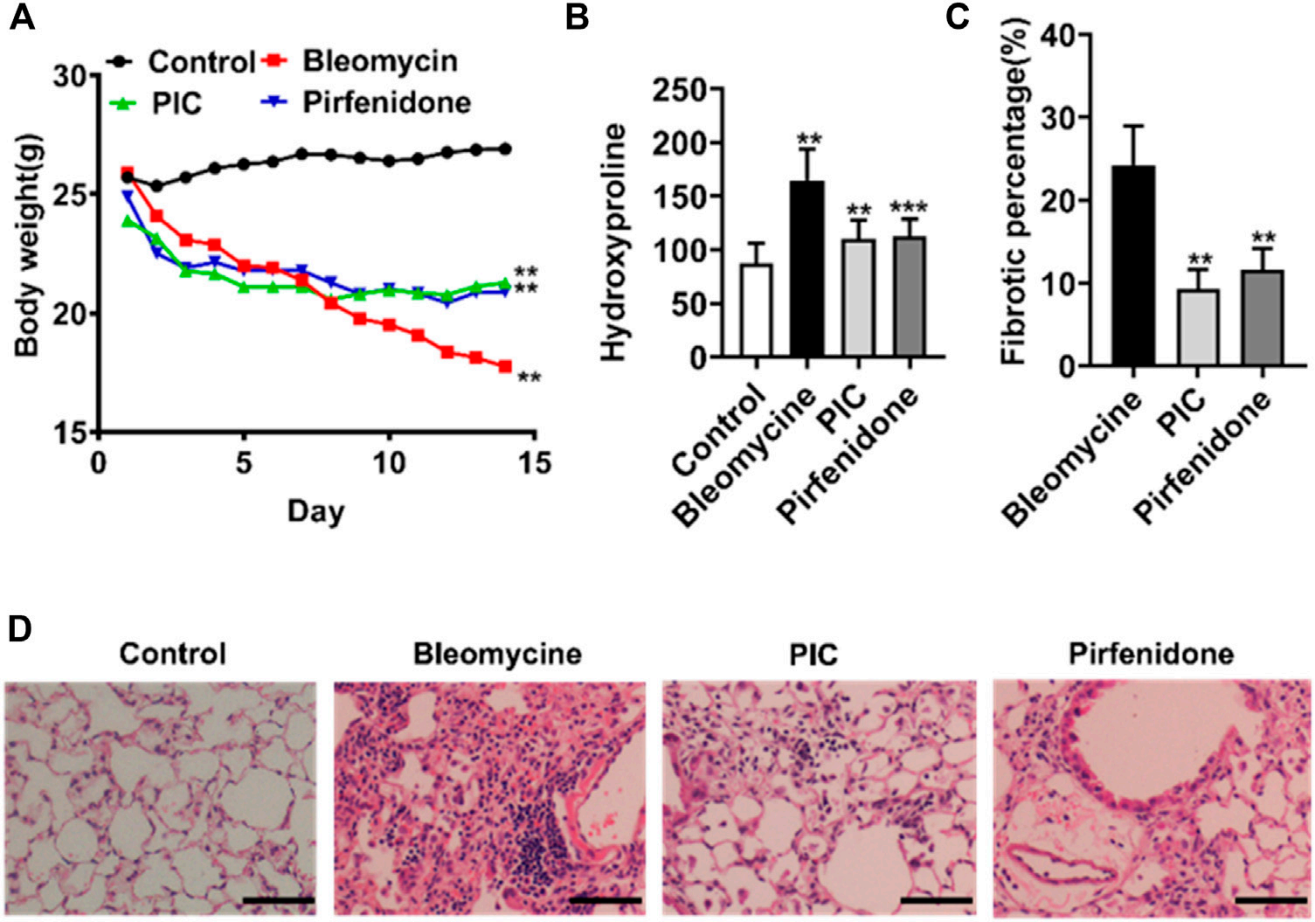
**(A)** Representative images showing the weight changes of therapeutic dosing of 10 mg/kg PIC in mice with the established bleomycin-induced pulmonary fibrosis model. **(B)** Hydroxyproline content of lung tissues from indicated groups of mice. **(C,D)** Hematoxylin-eosin staining and pulmonary fibrosis area of lung sections from the indicated groups of mice. Scale bars, 100 µm. Data are expressed as mean ± SD, n = 6. **p < 0.01.

In order to evaluate the potential anti-fibrosis effect of PIC, we established an experimental lung fibrosis model induced by BLM. Using this animal model, we found that treatment with PIC by daily gavage at a dose of 10 mg/kg body weight was well tolerated as no drug-related adverse events were observed. In addition, PIC treatment significantly improved BLM-induced weight loss in mice (Figure 4A). Compared with the BLM group, the hydroxyproline level was reduced significantly after treatment with PIC, suggesting a protective role of PIC in counteracting ECM accumulation (Figure 4B). As determined by hematoxylin-eosin staining of lung sections, the intratracheal injection of BLM led to the destruction of normal pulmonary architecture and the prominent proliferation of fibroblasts. Impressively, PIC significantly improved these pathological changes in mice, and the effect was better than pirfenidone (Figures 4C,D).

### PIC Reduces the Activation of Fibroblasts and Production of ECM in the BLM-Induced Pulmonary Fibrosis Model

**FIGURE 5:**
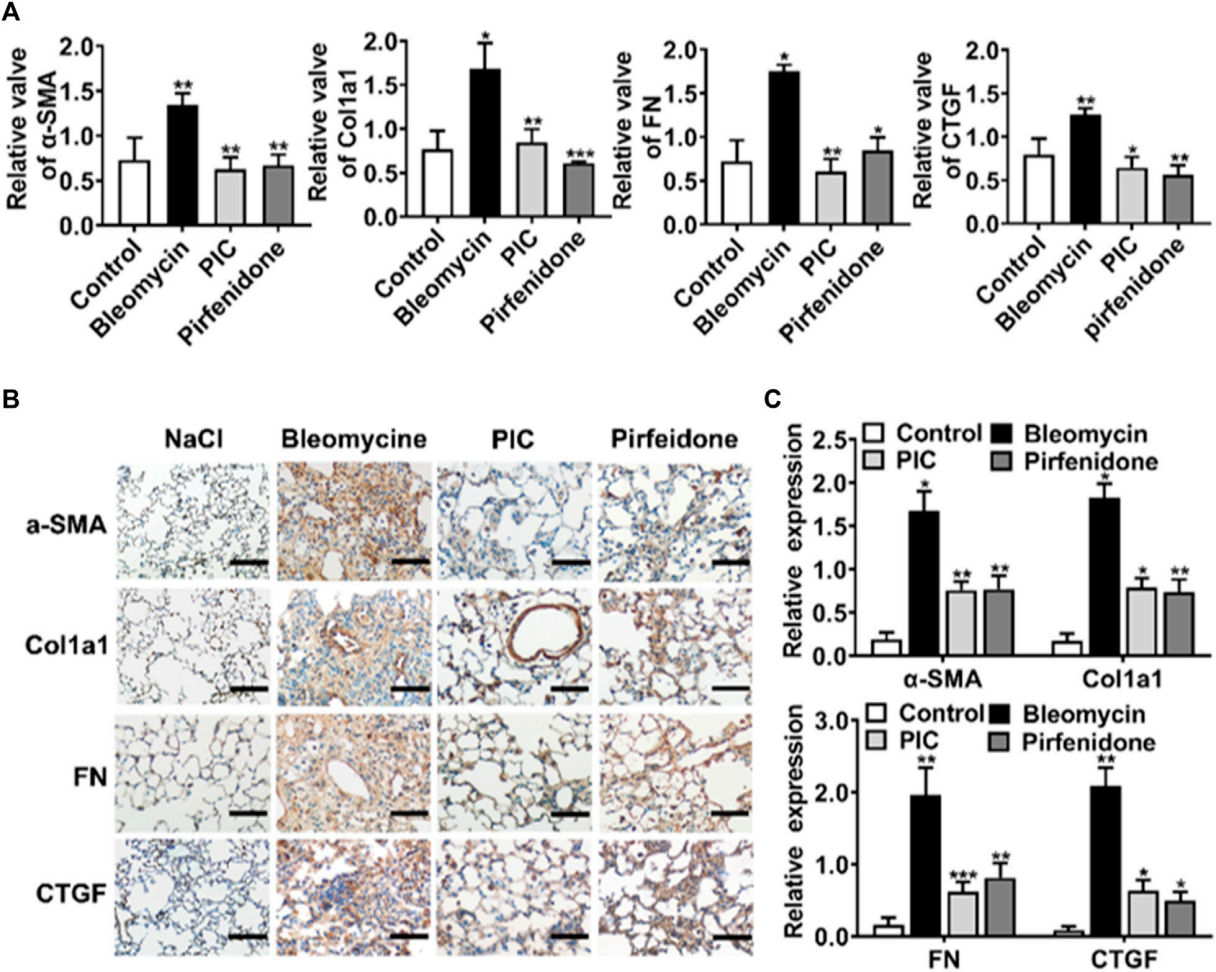
**(A)** mRNA expression levels of α-SMA, Col1a1, FN, and CTGF in lung homogenates of indicated groups. **(B)** Representative images showing immunohistochemical staining of α-SMA, Col1a1, FN, and CTGF of lung sections from the indicated groups of mice and images at 200× magnification. **(C)** Expressions of protein were performed by ImageJ in different groups. Data are expressed as mean ± SD, n = 6. *p < 0.05, **p < 0.01, and ***p < 0.001.

During the development of IPF, fibroblasts abnormally activated and accumulated, leading to excessive production of ECM in parenchymal cells. So we assessed the effect of PIC on the activation of myofibroblasts and ECM production in the BLM-induced pulmonary fibrosis model. The mRNA expression of reliable markers of activated myofibroblasts, such as α-SMA, Col1a1, FN, and CTGF, was substantially increased in mice treated with BLM. However, PIC significantly reduced the mRNA expression of these matrix components (Figure 5A). Next, lung sections were stained for α-SMA, Col1a1, FN, and CTGF. The results indicated that the expression of fibroblast activation makers was obviously ameliorated in the PIC-treated group (Figures 5B,C). Taken together, these results indicated that PIC ameliorated the excessive accumulation of myofibroblasts and ECM production in the BLM-induced pulmonary fibrosis model.

### PIC *via* the Smad3/ERK/P38 Signaling Pathway Regulated Autophagy *in Vivo*

**FIGURE 6:**
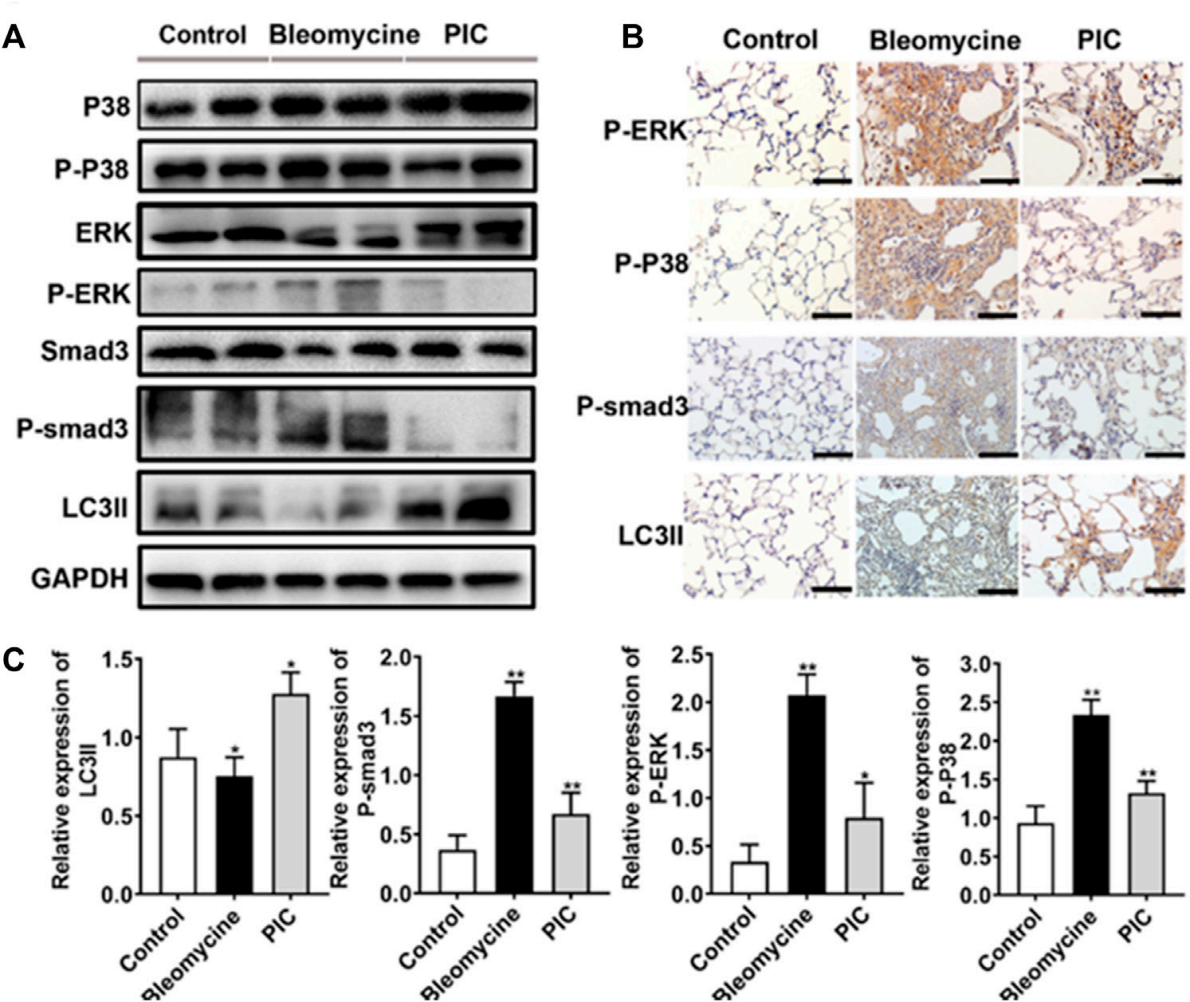
**(A)** Protein expression of LC3BII, P-Smad3, P-ERK and P-P38 in lung tissues of indicated groups. **(B)** Immunohistochemical staining of LC3BII, P-Smad3, P-ERK and P-P38 in lung sections of indicated groups. Scale bars, 100 μm. **(C)** Quantitative expression analysis of LC3II, P-Smad3, P-ERK and P-P38 in different groups. Data are expressed as mean ± SD, n = 6. *p < 0.05 and **p < 0.01.

To further confirm the anti-fibrotic mechanism of PIC, we examined the protein expressions of p-Smad3, p-ERK, p-p38, and LC3II in lung tissues by immunoblotting. Compared to BLM-treated mice, the results showed that the LC3II expression was enhanced, and p-Smad3, p-ERK, and p-p38 expressions were significantly decreased in PIC-treated mice (Figure 6A). Next, lung sections were immunostained for LC3II, p-Smad3, p-ERK, and p-p38. The results indicated that PIC-treated mice demonstrated enhanced expressions of LC3II and significantly reduced the expressions of p-Smad3, p-ERK, and p-p38 (Figures 6B,C). Taken together, these results indicated that PIC may improve pulmonary fibrosis by enhancing autophagy *via* inhibiting the Smad3/ERK/P38 signaling pathway. These results were consistent with *in vitro* studies.

## Discussion

IPF is a refractory fibrotic disease of the lung, and most patients deteriorate with a mortality of 50% in 2–3 years from diagnosis (Liu et al., 2018). With the outbreak of COVID-19, the emergence of complications like IPF among seriously sick patients made it one of the main problems that needed to be resolved and treated effectively (Kang et al., 2020). There is still an urgent need to develop more effective therapies for IPF patients. The major finding of our present study demonstrated that the anti-fibrotic effects of PIC may be due to several mechanisms, including direct pulmonary protective effects and autophagy (Figure 2). In the present work, we found that treatment of PIC obviously attenuated BLM-induced weight loss in mice and reduced the hydroxyproline content in the lung tissue. The HE staining also showed that the lung tissue structure of the PIC-treated group was more complete than the model group, and the degree of alveolitis/ and pulmonary fibrosis was lower than in the model group. These data showed that oral treatment of PIC attenuates bleomycin-induced pulmonary fibrosis in mice.

Autophagy is a catabolic process with “self-protective” functions that provides a mechanism under particular conditions (Diptiman et al., 2018). Promotion of autophagy is necessary and sufficient to inhibit fibroblast proliferation (Lin et al., 2018). Several compounds possess attenuated pulmonary fibrosis activity through autophagy-dependent mechanisms (Wan et al., 2018). In order to demonstrate autophagy induction activity between PIC and IPF, we evaluated the levels of LC3 and P62 expressions by Western blot. The PIC significantly upregulated the LC3II protein expression and decreased the P62 protein expression and decreased α-SMA and collagen type I expressions compared with TGF-β1 and CQ (autophagy inhibitor). Our findings suggested that PIC can promote autophagy in fibroblasts and reduce the number of pulmonary fibrosis effector-myofibroblasts.

Transforming growth factor-β (TGF-β) is a member of a family of polypeptides that plays central role in lung fibrogenesis (Aschner and Downey, 2016). TGF-β induction of fibroblast proliferation may be mediated through Smad3 pathways (Aschner and Downey, 2016), specifically through ERK and P38 activation (Wang et al., 2018). In addition, Smad3/ERK/P38 multiple signaling pathways regulate the occurrence of autophagy or mediate the activity of autophagy (Yang et al., 2020; Li et al., 2021). In this study, PIC plays a role in activating autophagy in the process of anti-fibroblast activation. Further mechanism studies demonstrated that PIC can promote autophagy *via* inhibiting the TGF-β1-Smad3/ERK/P38 signaling pathway, which leads to a decreased number of activated myofibroblasts. PIC significantly inhibited the expression levels of phosphorylated Smad3, ERK, and P38. The PIC treatment group significantly reduced the expressions of α-SMA, Col1a1, and FN mRNA. These results suggest that PIC by activating autophagy *via* inhibiting the TGF-β1/Smad3/ERK/P38 signaling pathway directly attenuates pulmonary fibrosis.

Anti-fibrotic therapies that are available or in development could have value in preventing severe COVID-19 in patients with IPF, have the potential to treat severe COVID-19 in patients without IPF, and might have a role in preventing fibrosis after SARS-CoV-2 infection (Pmga et al., 2020). PIC has many obvious advantages in exerting drug anti-fibrosis and reducing inflammation in various diseases (Peng et al., 2020). In this study, the inhibitors of the Smad3/ERK/P38 signaling pathway significantly promoted the level of autophagy and decreased the expressions of α-SMA, Col1a1, and FN. Based on the current evidence, it is suggested that PIC may target the Smad3/ERK/P38 signaling pathway to produce these therapeutic effects for PF, referring to supplementary data. However, future questions, with these comments in mind, need to be addressed, including the assessment of TGF-β levels in bronchoalveolar lavage fluid. In addition, 3MA, ATG5 siRNA, or Beclin 1 siRNA also should be used to further investigate in-depth the role of autophagy in PIC-treated cells stimulated by TGF-β1. In conclusion, the development and progress of PIC can provide a more effective clinical treatment approach for the sequelae of SARS-COV-2 pulmonary fibrosis and can also provide ideas for the utilization of TCM in the treatment of IPF.

## Data Availability

The original contributions presented in the study are included in the article/Supplementary Materials; further inquiries can be directed to the corresponding author.
